# Characteristics and Outcomes of Infectious Diseases Electronic COVID-19 Consultations at a Multisite Academic Health System

**DOI:** 10.7759/cureus.19203

**Published:** 2021-11-02

**Authors:** Kruti J Yagnik, Hala A Saad, Helen L King, Roger J Bedimo, Christoph U Lehmann, Richard J Medford

**Affiliations:** 1 Infectious Diseases, University of Texas Southwestern Medical Center, Dallas, USA; 2 Pediatrics, University of Texas Southwestern Medical Center, Dallas, USA

**Keywords:** infection prevention and control, infection control, infectious disease, virtual consult, covid-19 pandemic

## Abstract

Objective

The need for clinicians to access Infectious Diseases (ID) consultants for clinical decision-making support increased during the Coronavirus Disease 2019 (COVID-19) pandemic. Traditional ID consultations with face-to-face (FTF) patient assessments are not always possible or practical during a pandemic and involve added exposure risk and personal protective equipment (PPE) use. Electronic consultations (e-consults) may provide an alternative and improve access to ID specialists during the pandemic.

Methods

We implemented ID e-consult platforms designed to answer clinical questions related to COVID-19 at three academic clinical institutions in Dallas, Texas. We conducted a retrospective review of all COVID-19 ID e-consults between March 16, 2020 and May 15, 2020 evaluating characteristics and outcomes of e-consults among the clinical sites.

Results

We completed 198 COVID-19 ID e-consults at participating institutions. The most common e-consult indications were for 63 (32%) repeat testing, 61 (31%) initial testing, 65 (33%) treatment options, and 61 (31%) Infection Prevention (IP). Based on the e-consult recommendation, 53 (27%) of patients were initially tested for COVID-19, 45 (23%) were re-tested, 44 (22%) of patients had PPE precautions initiated, and 37 (19%) had PPE precautions removed. The median time to consult completion was four hours and 8 (4%) consults were converted to standard FTF consults.

Conclusion

E-consult services can provide safe and timely access to ID specialists during the COVID-19 pandemic, minimizing the risk of infection to the patient and health care workers, while preserving PPE and testing supplies.

## Introduction

The Coronavirus Disease 2019 (COVID-19) pandemic brought unprecedented circumstances for clinicians with little available evidence to guide clinical decision-making. The resulting uncertainty increased the demand for Infectious Diseases (ID) specialists to assist in patient care by providing prompt case evaluation and recommendations. Standard ID consultation requires a resource expensive, time-consuming face-to-face (FTF) encounter with the patient, which is often impossible or impractical during a pandemic. Electronic consultations (e-consults) are virtual consultative communications without direct physical contact between the consulting clinician and the patient [1]. The implementation of electronic health records (EHRs) in the vast majority of US hospitals addressed the basic requirements for e-consults by allowing ubiquitous access for the ID specialist to relevant patient data. 

E-consult platforms for specialty services have been successfully implemented in outpatient and inpatient environments [1-3]. Even prior to the COVID pandemic, telemedicine technologies were increasingly being used to deliver healthcare services because of their power to overcome distance barriers, improve access to primary care, reduce costs, improve education, reduce time to consultation, and improve outcomes [4-7]. In addition, e-consults improve access to overburdened specialty clinics, reduce FTF referrals, and improve provider-to-provider communication [8,9]. Outpatient e-consults are linked to improved patient satisfaction, high favorability among primary care physicians (PCPs), and superior patient safety [10]. Asynchronous ID e-consults for hospitalized patients provide effective specialist services to remote hospitals lacking access to telemedicine equipment or FTF specialist services. Compared with matched controls, inpatient ID e-consults resulted in a 70% reduction in 30-day mortality for patients and a decrease in 30-day readmission [11].

An ID e-consult platform offers benefits during a pandemic with the potential to minimize unnecessary healthcare worker exposure to COVID-19, decrease the use of personal protective equipment (PPE), and provide prompt patient care recommendations. We sought to assess the impact of an ID e-consult platform in response to consult requests related to COVID-19 at three clinical institutions in Dallas, Texas.

This article was previously presented as a meeting poster presentation at the IDWeek 2020 on October 21, 2020.

## Materials and methods

Leveraging an existing e-consult platform in our EHR, COVID-19 ID e-consults were implemented to respond to consult requests at three hospital systems affiliated with the University of Texas Southwestern Medical Center: Clements University Hospital (CUH), Parkland Health and Hospital System (PHHS), and the VA North Texas Health Care System (VA). E-consults were inpatient-only at the VA and PHHS. CUH had both inpatient and outpatient e-consults. These e-consults were exclusively an EHR review of the patient’s chart. No video/telephone communications were used to contact the hospitalized patients.

We chose the institutions for the diversity of patients and the payment models they represent. CUH mainly provides care to insured patients, while PHHS is a county safety-net institution where most patients are under- or uninsured. The VA cares for veterans and represents yet another segment of patients with a third payment model. We performed a nine-week retrospective chart review of COVID-19 e-consults completed between March 16, 2020 and May 15, 2020, at the three institutions in North Texas.

PHHS, with a capacity of 870 hospital beds, had approximately 72,341 admissions from January 1, 2019 to December 31, 2019, with 241,968 ER visits. PHHS has an established outpatient ID e-consult platform and had responded to a total of 271 outpatient ID e-consults from March 1, 2019 to February 29, 2020. During the same nine-week time period in 2019 (prior to COVID-19), a total of 46 outpatient ID e-consults were completed from March 16, 2019 to May 17, 2019. The North Texas VA has a capacity of 853 hospital beds (including the spinal cord injury center and community living center) and had approximately 65,515 admissions from January 1, 2017 to December 30, 2017 (the most recent annual report that was available). CUH with 608 hospital beds had approximately 28,677 admissions from January 1, 2019 to December 31, 2019.

**Figure 1:**
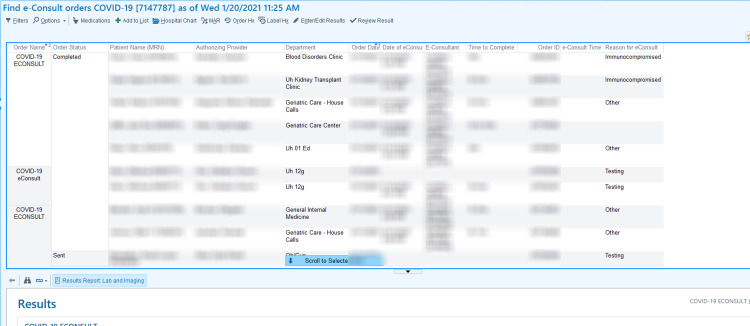
Electronic Health Record reporting dashboard.

We collected data on patient characteristics including demographics, comorbid conditions, severe acute respiratory syndrome coronavirus 2 (SARS-CoV-2) positivity at the time of e-consult, and place of residence (e.g., skilled nursing or rehabilitation facility). We created reporting dashboards in the EHR (Epic Systems Corporation; Verona, WI) at two institutions (CUH and PHHS) to track e-consult characteristics including ordering department, the reason for e-consult, and time to completion (Figure 1). At the VA (Veterans Health Information Systems and Technology Architecture [VistA]), we extracted the same data by chart review for all completed e-consults. Outcomes and recommendations of e-consults were manually reviewed by two authors with a third author adjudicating discrepancies and included initiation or removal of PPE, a recommendation to test or retest for COVID-19, and conversion of the e-consult to a formal ID consult. 

We analyzed continuous variables using one-way ANOVA and Kruskal-Wallis tests with posthoc analysis using Tukey's honestly significant difference and Dunn tests, respectively. Given the non-Gaussian populations, categorical variables were analyzed using Chi-squared and Fisher’s exact test with posthoc Bonferroni correction. Alpha level of significance was set a priori at 0.017 (0.05/3) to determine significance among the three groups and all hypothesis testing was two-sided. All statistical analyses were performed using RStudio (version 1.3.959).

This study was approved by the Institutional Review Boards at Clements University Hospital, Parkland Health and Hospital System, and the North Texas VA Medical Center.

## Results

We completed 198 COVID-19 ID e-consults at the three institutions during our nine-week study period with the first e-consult performed at the VA on March 20, 2020. The mean patient age (SD) was 55 years (15.9) (Table 1). Mean ages at CUH and PHHS were significantly lower than at the VA (CUH-VA [p < 0.01], PHHS-VA [p < 0.01]), but there were no differences between CUH and PHHS (p = 0.98). Our cohort had 122 (62%) males with an expected higher proportion of males at the VA compared to CUH (p < 0.01) and PHHS (p < 0.01). Overall, 79 (40%) of patients were White, 71 (36%) Hispanic, 42 (21%) Black, and 6 (3%) Asian (Table 1). There were also significant differences noted in the number of Whites (p < 0.01), Hispanics (p < 0.01), and Asians (p < 0.01) among the three hospitals but not in the number of Blacks (p = 0.03).

**Table 1:** Patient characteristics of ID e-consult at each clinical site ID: Infectious Diseases; e-consults: Electronic consultations; PCR: Polymerase chain reaction; COPD: Chronic obstructive pulmonary disease; CUH: Clements University Hospital; VA: Veterans Affairs.

		Clinical Site			
Patient characteristics	CUH n (%)	Parkland n (%)	VA n (%)	Total n (%)	P-value
Sex					
Female	28 (41)	44 (51)	4 (9)	76 (38)	p < 0.01
Male	40 (59)	42 (49)	40 (90)	122 (62)	p < 0.01
Age (mean [SD])	53 (16)	52 (15)	65 (13)	55 (16)	p < 0.01
Race/Ethnicity					
White	34 (50)	19 (22)	26 (59)	79 (40)	p < 0.01
Black	15 (22)	12 (14)	15 (34)	42 (21)	p = 0.03
Hispanic	13 (19)	55 (64)	3 (7)	71 (36)	p < 0.01
Asian	6 (9)	0 (0)	0 (0)	6 (3)	p < 0.01
Comorbidities					
Cardiac Condition	13 (19)	42 (49)	34 (77)	89 (45)	p < 0.01
Diabetes mellitus	12 (14)	40 (47)	17 (39)	69 (39)	p < 0.01
COPD or Asthma	8 (12)	11 (13)	11 (25)	30 (15)	p = 0.12
End-stage renal disease	3 (4)	6 (7)	2 (45)	11(5)	p = 0.85
Severe obesity	0 (0)	6 (7)	3 (7)	9 (4.5)	p = 0.04
Liver disease	3 (4)	5 (6)	6 (14)	14 (7)	p = 0.18
Immunocompromised	20 (29)	22 (26)	12 (27)	54 (27)	p = 0.74
Resident of skilled nursing facility	0 (0)	12 (14)	14 (32)	26 (13)	p < 0.01
SARS-CoV-2 PCR positive	20 (29)	36 (42)	19 (43)	75 (38)	p = 0.20

Patient comorbidities (Table 2) included: 89 (45%) with a heart condition, 77 (39%) with diabetes mellitus, 54 (27%) with an immunocompromising condition, 30 (15%) with asthma, 14 (7%) with liver disease, 11 (5%) with end-stage renal disease (ESRD), and 9 (4.5%) with morbid obesity (Table 1). Additionally, 26 (13%) of patients were residents of a long-term care facility. At the time of the e-consult, 75 (38%) of patients tested positive for COVID-19 by SARS CoV-2 PCR, while all others tested negative or were presumed negative (Table 1).

**Table 2:** Definition of comorbidities listed for patient characteristics.

Comorbidity	Definition
Cardiac condition	Hypertension, heart failure (systolic, diastolic, or both), coronary artery disease, or congenital heart disease
End-stage renal disease (ESRD)	On renal replacement therapy: hemodialysis (HD), peritoneal dialysis (PD), or continuous renal replacement therapy (CRRT)
Severe obesity	BMI ≥ 40
Immunocompromised	HIV/AIDS, active malignancy, transplant recipient, or on chronic immunosuppressive medications (steroids, immunomodulators, etc.)
Liver disease	Cirrhosis, hepatitis, tumor (benign or malignant), autoimmune liver disease, or genetic liver disease

The department ordering most of the inpatient e-consults was Internal Medicine 148 (85%), followed by the surgical services, neurology, and OB-GYN (Table 3). The ability to request an ambulatory COVID-19 e-consult was available at CUH during the study period and represented 23 (34%) of total e-consults at this institution. This service was not available at PHHS or the VA. In the ambulatory setting, Internal Medicine was also the most frequently ordering department (10 [43%]) followed by the surgical services (Table 3).

**Table 3:** ID e-consult characteristics at each clinical site. IQR: Interquartile range; OB/GYN: Obstetrics and Gynecology; ID: Infectious Diseases; e-consult: Electronic consultations; FTF: Face-to-face.

E-consult characteristics	CUH N (%)	Parkland N (%)	VA N (%)	Total N (%)	P-value
Total number of consults	68	86	44	198	
Ordering Department					
Inpatient	45 (66)	86 (100)	44 (100)	175 (88)	
Internal Medicine	34 (76)	71 (83)	43 (98)	148 (85)	
Medical ICU	1 (2)	2 (2)	1 (2)	4 (2)	
Surgical Service	0 (0)	5 (6)	0 (0)	5 (3)	
Oncology	3 (6)	1 (1)	0 (0)	4 (2)	
Neurology	4 (9)	1 (1)	0 (0)	5 (3)	
OB/GYN	2 (4)	3 (3)	0 (0)	5 (3)	
Psychiatry	1 (2)	1 (1)	0 (0)	2 (1)	
Other Inpatient	0 (0)	2(2)	0 (0)	2 (1)	
Outpatient	23 (34)	0 (0)	0 (0)	23 (12)	
Internal Medicine	10 (43)			10 (43)	
Medical ICU	0 (0)			0	
Surgical Service	6 (26)			6 (26)	
OB/GYN	0 (0)			0	
Psychiatry	0 (0)			0	
Oncology	4 (17)			4 (17)	
Neurology	3 (13)			3 (13)	
Other	0 (0)			0	
Time to Completion (median in (hours), IQR)	4 (2-7.5)	2 (1.25-4.75)	5.5 (4-9.25)	4 (2-7)	p < 0.01
Converted to FTF (formal consult)	0 (0)	8 (9)	0 (0)	8 (4)	p < 0.01

The median (interquartile range [IQR] time to e-consult completion was 2h (1.25-4.75), 4h (2-7.5), and 5.5h (4-9.25) at PHHS, CUH, and the VA, respectively. The shorter time to completion at PHHS was significant when compared to CUH (p < 0.01) and the VA (p < 0.01), but there was no significant difference between CUH and the VA (p = 0.24).

**Figure 2:**
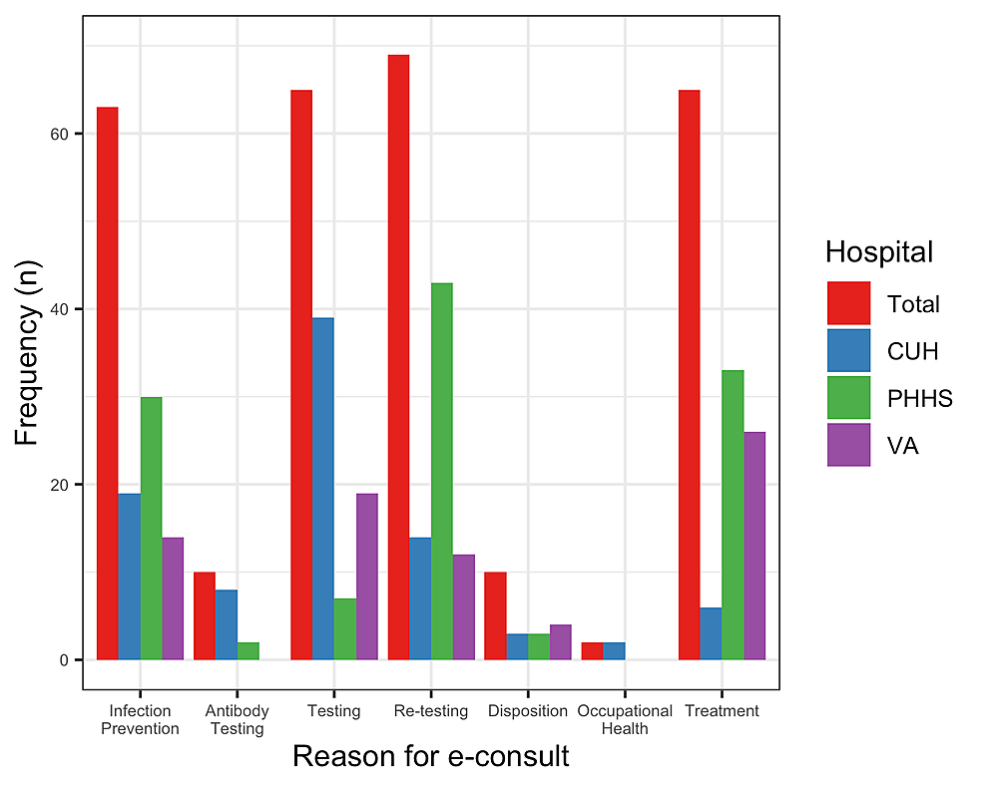
Reasons for e-consult, by hospital. e-consults: Electronic consultations; CUH: Clements University Hospital; PHHS: Parkland Health and Hospital System; VA: Veterans Affairs.

**Figure 3:**
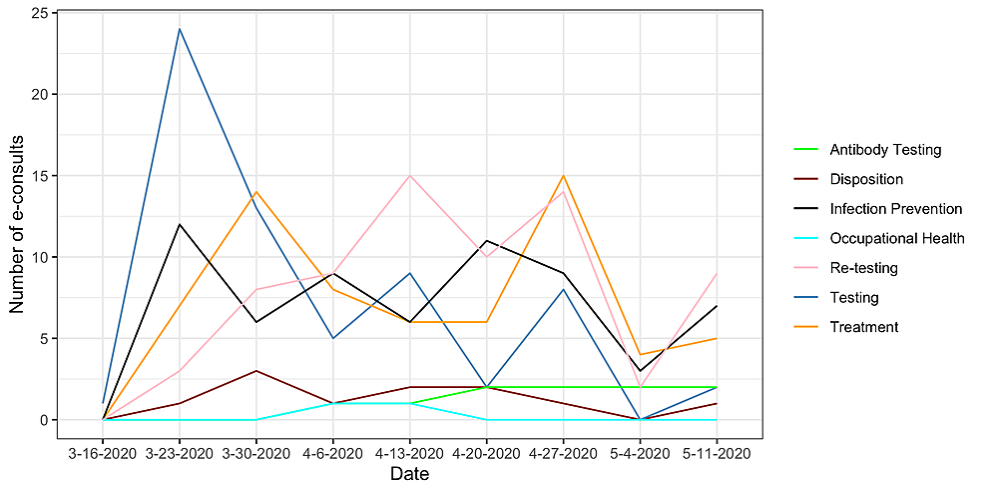
Reasons for e-consult, by week. e-consults: Electronic consultations.

The most common reasons for e-consult included: 69 (35%) need for repeat testing followed by 65 (33%) initial testing, 65 (33%) treatment, and 63 (32%) infection prevention and control (IPC). Antibody testing (10 [5%]), disposition (10 [5%]), and occupational health (1 [2%]) related questions were the least common reasons for an e-consult. There was also a significant inter-institutional variation with providers at PHHS most commonly asking repeat testing questions 43 (62%), (p < 0.01) while initial testing questions were more common (p < 0.01) at CUH 39 (60%) and the VA 19 (30%) (Figure 2). Testing questions were predominantly asked in the first six weeks of our study period, peaking at two weeks and trailing off, while other categories remained consistent throughout the study period (Figure 3).

**Figure 4:**
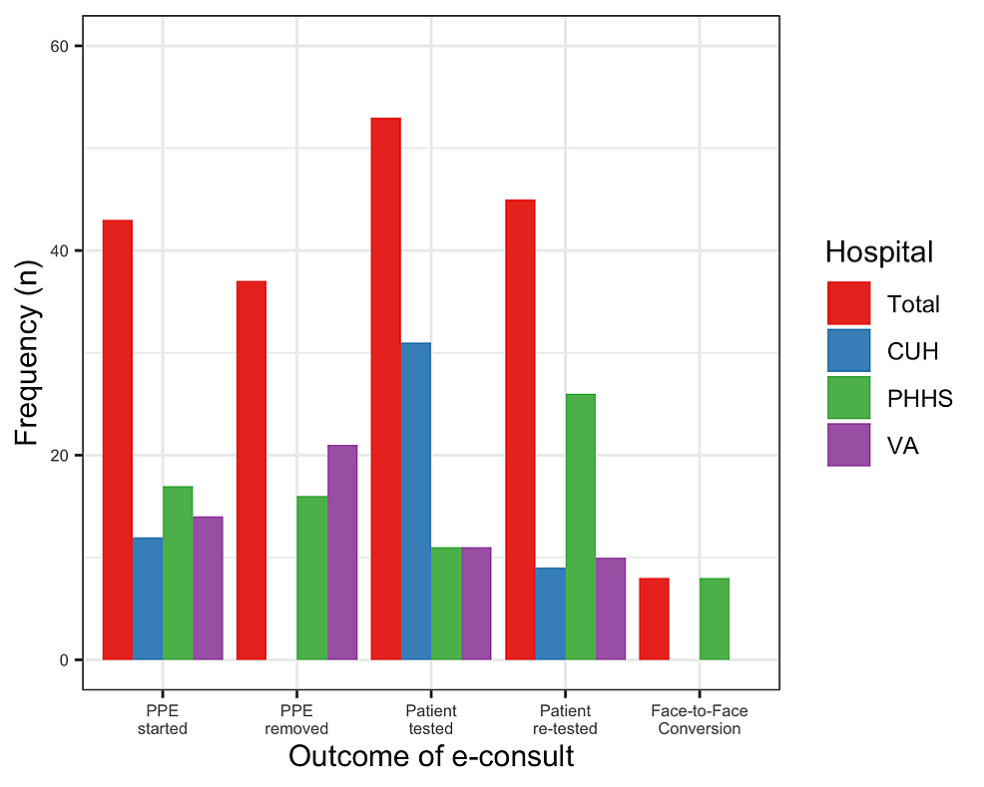
Outcomes of e-consult, by hospital. e-consults: Electronic consultations; CUH: Clements University Hospital; PHHS: Parkland Health and Hospital System; VA: Veterans Affairs.

At the discretion of the ID clinicians, 8 (4%) of e-consults were converted to a standard FTF consult. Based on the e-consult recommendation, 53 (27%) of patients were tested, 45 (23%) were re-tested, 44 (22%) of patients had PPE precautions initiated, and 37 (19%) had PPE precautions removed (Figure 4). There were inter-institutional variations in recommendations and patients were more likely to be tested at CUH 31 (58%), re-tested at PHHS 26 (58%), and have PPE initiated at PHHS 17 (40%). Finally, there was a significant difference in PPE removal among the three institutions, with removal more common at the VA (21 [57%], p < 0.01).

## Discussion

The rapid growth of the COVID-19 pandemic and the associated strain on the healthcare system and its resources required clinicians to quickly adapt to new technologies and models of care. We provide a foundational analysis of the role of e-consults in the management of COVID-19 by describing the characteristics and outcomes of e-consults performed at three clinical institutions in Dallas, Texas. We demonstrate that an e-consult platform can be utilized in ambulatory and inpatient settings to provide specialist input in a timely fashion, promoting safe and equitable care early in a pandemic. 

As illustrated by the diverse patient populations and comorbidities, e-consults offer a flexible framework to answer a variety of clinical inquiries. Moreover, diversity is likely a reflection of the different care settings represented by private, county, and veterans' hospitals, CUH, PHHS, and the VA, respectively. Early in the pandemic, the majority of questions were related to the need for testing; however, as testing became readily available, new questions arose related to previous disease (antibody testing) and treatment options. Other question categories remained relatively stable throughout the nine-week period. We hypothesize that lengthening our study period would demonstrate the dynamics of a pandemic and resulting consult questions as new and evolving information was incorporated into clinical care. 

The benefits of ID e-consults have been previously studied [12] and evaluation has been extended to the COVID-19 pandemic [3]. An e-consult offers a formalized way to document the ID specialist’s recommendations in a systematic and transparent manner, to increase productivity in a time of increased demand, to minimize ‘curbside’ conversations while reducing PPE use, and to allow those, who enter the chart to understand clinical reasoning [4]. E-consults are an essential form of communication when there is significant infection control risk and uncertainty, like the beginning stages of an outbreak. The recommendations of ID specialists promoted diagnostic stewardship by decreasing inappropriate tests, which continue to be a scarce resource. E-consults also promoted appropriate infection prevention and control practices by initiating and removing PPE when necessary. Although most major university health systems have dedicated healthcare infection prevention programs to address common infection prevention questions, very early in the pandemic these questions were frequently addressed by inpatient ID consult services. Once appropriate protocols and procedures were in place at all three hospital sites in our study, questions regarding infection control/prevention decreased and were then addressed mostly by the dedicated infection prevention teams.

Although e-consults are asynchronous in nature, timely responses are necessary for their successful adoption by the consulting clinician. With a median time to completion of four hours, recommendations were given in a prompt manner, allowing providers to make efficient operational and clinical decisions. The shorter median time to completion at PHHS of two hours likely reflects their unique use of the e-consult to triage COVID-19 patients. Additionally, only a small subset of consults was converted to FTF interactions, highlighting their effectiveness by appropriately answering COVID-related questions. The most common reasons for e-consults to be converted to FTF interactions were: requiring additional history from the patient, needing a full history and physical exam for a complicated case, or needing to examine a patient due to the possibility of skin/soft tissue infection or rash.

While we were successful in implementing e-consults across disparate health systems and EHRs, there are significant barriers that often prevent widespread dissemination. One of the main factors is the lack of a standardized reimbursement structure [13]. In the US, dramatic changes in the Centers for Medicare & Medicaid Services telehealth regulations allowed for rapid adoption of these technologies; however, reimbursement from private insurers remains varied [13,14]. Furthermore, without dedicated information technology resources, the building and maintenance of the e-consult platforms are challenging.

Limitations

Our study has several limitations. First, we used a retrospective study design, and there are inherent limitations associated with chart abstraction and data collection. Second, we evaluated a single academic health system and our results may not be broadly applicable to other health systems. However, we do believe that including three diverse hospital types and patient populations and three EHRs from two vendors may help in increasing generalizability. Also, we only evaluated the first nine weeks of the pandemic in our local region and therefore questions asked and outcomes of e-consults beyond this timeframe may show different results. Furthermore, this is a descriptive study and there is no comparison between e-consults and FTF consults for patient satisfaction, provider satisfaction, and clinical outcomes.

## Conclusions

Our findings suggest that e-consults are an ideal medium to provide timely ID recommendations in the early stages of a pandemic while fostering an environment that promotes safety and diagnostic and resource stewardship. E-consults can serve a diverse patient population with differing comorbidities, minimize waste of testing reagents and PPE, and ensure appropriate healthcare worker protective precautions. In summary, we believe e-consults will continue to play a vital role in the management of patients through this pandemic and can be leveraged for future ones.
